# Infodemic Preparedness and COVID-19: Searching about Public Health and Social Measures Is Associated with Digital Health Literacy in University Students

**DOI:** 10.3390/ijerph191912320

**Published:** 2022-09-28

**Authors:** Rafaela Rosário, Inês Fronteira, Maria R. O. Martins, Cláudia Augusto, Maria José Silva, Melanie Messer, Silvana Martins, Ana Duarte, Neida Ramos, Katharina Rathmann, Orkan Okan, Kevin Dadaczynski

**Affiliations:** 1 School of Nursing, University of Minho, 4710-057 Braga, Portugal; 2 Health Sciences Research Unit: Nursing (UICISA: E), Nursing School of Coimbra (ESEnfC), 3000-232 Coimbra, Portugal; 3 Nursing Research Centre, University of Minho, 4710-057 Braga, Portugal; 4 Global Health and Tropical Medicine, Institute of Hygiene and Tropical Medicine, NOVA University of Lisbon, 1349-008 Lisboa, Portugal; 5 Department of Nursing Science II, Faculty I, Trier University, 54296 Trier, Germany; 6 Department of Health Science, Fulda University of Applied Sciences, 36037 Fulda, Germany; 7 Department of Sport and Health Sciences, Technical University of Munich, 80992 Munich, Germany; 8 Center for Applied Health Science, Leuphana University of Lueneburg, 21335 Lueneburg, Germany

**Keywords:** digital health literacy, public health and social measures, infodemic, COVID-19

## Abstract

We aimed to evaluate the associations between information searching about public health and social measures (PHSM) and university students’ digital health literacy (DHL) related to the new coronavirus (SARS-CoV-2) and COVID-19. Methods: This cross-sectional study included 3,084 Portuguese university students (75.7% females), with an average age of 24.2 (SD = 7.5). Sociodemographic data, DHL questionnaire and online information concerning PHSM were gathered. Cox proportional hazards models were performed. Results: Students who searched for personal protective measures achieved in shorter time sufficient “evaluating reliability” (HR = 1.4; 95% CI = 1.1; 1.7) and “determining relevance” (HR = 1.5; 95% CI = 1.2; 1.8). Searching for surveillance and response measures was associated with sufficient “determining relevance” (HR = 1.4; 95% CI = 1.1; 1.9). Finally, those students who searched for environmental, economic and psychosocial measures achieved in shorter time “determining relevance” (HR = 1.2; 95% CI = 1.0; 1.4). Conclusions: Searching for PHSM was significantly associated with an increased likelihood of achieving sufficient DHL subscales in a shorter time. Further studies are needed, including developing strategies to increase the availability of high-quality information concerning public health and social measures and to improve (digital) health literacy.

## 1. Introduction

The world is facing an unprecedented public health and social crisis. There are imperative efforts to learn from the pandemic response [1]. Future pandemic preparedness is being developed in order to ensure that people are better prepared to face a next pathogen [1,2]. Public health and social measures (PHSM) (e.g., personal protective measures, environmental measures, surveillance and response measures) have been crucial to control outcomes [3].

We know that information on COVID-19 and related topics is abundant and available through a full range of digital media and technologies [4]. However, we face an overabundance of (mis/dis)information and its rapid spread, also known as the infodemic [5]. The infodemic can result in confusion and risk-taking behavior that is harmful to health [6,7]. Therefore, identifying the PHSM information-seeking behavior about COVID-19 and its associations with health literacy can provide valuable insights into the factors that influence people’s health behavior [8] and guide the long-term direction when addressing future threats.

The rapidly evolving situation of COVID-19 leads to citizens’ inability to filter, follow and integrate the quickly changing facts as well as the information and demands published daily [8,9]. Moreover, health information on the Internet is often complex and even conflicting. Therefore, people need to be equipped with the skills, knowledge and motivation to access, navigate, understand and evaluate health information, and to use it to make informed decisions and transfer it into everyday health behaviors and practices [10]. Therefore, (digital) health literacy is vital during a pandemic [11,12].

According to the Digital Economy and Society Index (DESI), the most active Internet users are young individuals (97% aged between 16 and 24) with a high level of formal education (97%) and students (98%) [13]. Still, there has been scarce research about the information-seeking behavior of young adults, notably university students, and the DHL when searching, finding, evaluating and integrating COVID-19-related information into everyday life. A recent study found that higher levels of health literacy in medical university students are associated with less fear of COVID-19 than those with low health literacy levels [14]. Moreover, initial findings from web-based surveys of university students suggest significant associations between information-seeking behavior and digital health literacy in Portugal [15], Germany [16], Denmark [17] and East and South-East Asia [18]. However, students’ online information queries in the context of SARS-CoV-2 and COVID-19, and the determinants of digital health literacy (DHL), are new, and studies are just beginning to be published. When preparing for future pandemics, addressing the information-seeking behavior and DHL is essential for successfully procuring resources and measures ready to be transformed, adapted and integrated into practice [19]. Hence, this study aims to evaluate the associations between information searching about PHSM and university students’ DHL related to the SARS-CoV-2 and COVID-19 during the first wave of the pandemic in Portugal and associated university closures.

## 2. Materials and Methods

### 2.1. Participants

The current study is part of the COVID–HL research consortium, a network composed of researchers from 45 countries conducting a DHL survey concerning coronavirus and COVID-19 among university students (https://covid-hl.eu, [20]). The Portuguese study comprised a total of N = 3,084 university students (75.7% of whom were females) participating in the survey. Data was collected from 28 April, with 24,141 of COVID-19 confirmed cases, to 8 June 2020, with 34,885 COVID-19 confirmed cases. All Portuguese universities from the mainland and the archipelagos of Azores and Madeira were invited to participate in the online survey using the platform survey monkey. All students completed the informed written consent form before starting the study. The Ethics Commission for Life Sciences and Health Research approved the study (number CEICVS 020/2020).

### 2.2. DHL Related to Coronavirus and COVID-19

The questionnaire used in this study has been developed by Dadaczynski and colleagues based on existing validated scales [20]. DHL used a sequence of questions concerning how easy students found it to search for and add their own content and determine the reliability and relevance of information relating to coronavirus and COVID-19. These questions were taken from the DHL instrument [12] and amended to COVID-19 context. The DHL was adapted to Portuguese [21] using the subscales of the DHL instrument [12]. Each subscale included three items to be answered on a four-point likert scale (e.g., 1 = very difficult, 4 = very easy). The subscales were as follows: (i) online information searching on coronavirus, (ii) adding self-generated content, (iii) evaluating the reliability of coronavirus information and (iv) determining personal relevance of coronavirus information. A mean value was calculated for each subscale. Two subgroups were created using the median split (limited versus sufficient DHL) in the additional analysis.

### 2.3. Online Information about Public Health and Social Measures (PHSM)

Students were asked to indicate the specific topics they were searching for in the context of the new coronavirus and COVID-19. The assessment was based on a list of nine topics developed by Dadaczynski and colleagues [20], with yes or no answers (please see Supplementary Materials Figure S1). The topics were further adapted and analyzed according to the considerations provided by the World Health Organization PHSM [22]. Briefly, we used the following measures: (i) personal protective (e.g., individual measures to protect against infection, current situation assessments and recommendations and physical distancing measures (e.g., restrictions); (ii) surveillance and response (e.g., the current spread of the virus, transmission routes of the coronavirus and symptoms of COVID-19); and (iii) environmental, economic and psychosocial (e.g., hygiene regulations, economic and social consequences of the coronavirus, and dealing with psychological stress caused by the coronavirus). Each measure was computed as the sum of the topics and further analyzed as two categories: “no” (did not search) and “yes” (explored at least one of the topics).

### 2.4. Other Measurements

Subjective social status was assessed based on the MacArthur Scale [23], which uses a ladder (10 points-steps) to represent the self-perceived socioeconomic position of students.

Students’ scientific field was assessed according to the revised classification of science and technology (fos) in the Frascati Manual [24]. Students were also asked about the degree they were pursuing, including bachelor’s, integrated master’s, master’s, PhD, and others.

### 2.5. Data Analysis

Descriptive statistics were used to explore item-specific normality, and participant characteristics are presented as means, standard deviations (SD) and percentage (%).

Bivariate differences were analyzed using Mann–Whitney and chi-squared tests. Subsequently, associations between online information about public health and social measures and DHL related to coronavirus and COVID-19 were analyzed using time-to-event analysis under the presence of competing determinants. The hazard ratios (HRs) and 95% confidence intervals (CIs) for the DHL subscales according to online information about PHSM were calculated using multivariate Cox proportional hazards models. The interpretation for the hazard ratio means that the group of interest comparing to the reference group is likely (HR > 1) or less likely (HR < 1) to have a shorter time-to-event (i.e., to achieve sufficient DHL subscales) [25].

As potential confounders, we included any variables hypothesized as affecting DHL. This includes sex, age, subjective social status, course, and study degree. The proportional hazard assumption was analyzed using log–log plots and Schoenfeld’s residuals [26]. There was no violation of the proportional hazard assumption. Data analyses were performed using SPSS, version 28.0 (IBM, SPSS Inc. Chicago, IL, USA), considering a level of significance of 0.05.

## 3. Results

Most participants were students of the social sciences (36.5%) and were enrolled in bachelor’s degree programmes (50.7%). Participating male students were significantly older, belonged mainly to engineering sciences, and pursued higher study degrees than female students (see Table 1).

Being male was significantly associated with sufficient DHL related with COVID-19 in two subscales, “adding self-generated content” (χ^2^(1) = 7.2, *p* = 0.007) and “determining relevance” (χ^2^(1) = 4.9, *p* = 0.027) when compared to being female. Furthermore, low subjective social status was associated with a limited ability to determine the relevance of corona-related health information (χ^2^(1) = 6.6, *p* = 0.010), see Table 2. No significant differences by course and degree of study were found for any of the variables measured.

After adjusting for differences in sex, age, subjective social status, course and degree of study, those students who searched for personal protective measures were more likely to have a shorter time to achieve a sufficient “evaluating reliability” of information concerning COVID-19 (HR = 1.4; 95% CI = 1.1; 1.7) and “determining its relevance” (HR = 1.5; 95% CI = 1.2; 1.8). Those who searched for surveillance and response measures had a 1.4-fold (95% CI = 1.1; 1.9) increased likelihood of reporting in a shorter time sufficient DHL in the subscale “determining relevance”. Those who searched for environmental, economic and psychosocial measures had a 1.2 fold (95% CI = 1.0; 1.4) increased likelihood of reporting in a shorter time sufficient DHL in the subscale “determining relevance”. (Table 3).

## 4. Discussion

The searching of online information about PHSM was significantly associated with an increased likelihood of achieving sufficient DHL in the subscale “determining relevance” in a shorter time in university students. Moreover, those who searched for personal protective measures achieved a sufficient “evaluating reliability” of information concerning COVID-19 in a shorter time. These results are particularly relevant for preparedness for future pandemics.

Current findings are following those of Rovetta et al. [9], who report that search queries about public health issues increased as the number of cases of COVID-19. The current study was conducted during the early stages of the pandemic in Portugal, when the information provided by official sources, social media and others, the so-called “supply-side” [8], was mainly about COVID-19 (e.g., the number of new cases, washing hands, physical distance, staying at home). In addition, people’s compliance with prevention measures was considered high, likely because the information associated with these measures is of lower complexity than other health or disease information [27]. However, this might have changed over time, for example, in a subsequent wave, or when people have other challenges, namely, those related to the socioeconomic impact of the pandemic or psychological stress [27,28]. Furthermore, the Internet and, especially, social media play a crucial role in the rapid and diffuse growth of misinformation, fake news, conspiracy theories or others, which might contradict governments and public health recommendations [29]. Therefore, skills for navigating online information are considered decisive during the COVID-19 infodemic. Furthermore, social support and the increase in the visibility and understanding of reliable sources may also mitigate the effects of digital inequalities [30]. Although DHL is critical, other inequalities, such as access to computers/Internet, and “technology/computer literacy” may be of similar or higher importance when searching for online information.

Overall, university students perceive themselves as having adequate DHL levels, scoring mainly in the third and fourth quartile of the response range. This result is similar to the findings generated in Germany [28], England [31] or Denmark [17] of the COVID–HL research consortium. In the current study, the greatest challenge is adding self-generated content about coronavirus information, where most of the students (72.1%) were considered to have limited health literacy. It is possible that a greater amount of information is available in different sources [32] because knowledge related to coronavirus and COVID-19 is poorly secured and alters substantially throughout time, making students more self-critical and scoring less in this subscale. Furthermore, female students report having more difficulties adding self-generated content (e.g., on forums or social media) and evaluating the reliability of online coronavirus information compared to male students. Similarly, female students may be more critical about the information and scored lower on these subscales. Nevertheless, there is no clear evidence about the associations between gender and health literacy [33,34,35,36,37]. Since low health literacy is associated with a lower socioeconomic condition [33,37,38]—even in the context of online health information the associations of DHL with socioeconomics remain significant [39]—we included a proxy measure of subjective social status [23] as a potential confounder along with education (e.g., the degree of studies and course).

Searching for all the evaluated PHSM was associated with a higher likelihood of achieving sufficient health literacy in a shorter time in the subscale “determining relevance”. It is possible that searching for PHSM allowed for the adoption of decision-making into daily life more rapidly. The World Health Organization underscored that PHSM acceptability and feasibility was determined through participatory approaches and engagement with the community, so that the likelihood of adherence was maximized [22]. It is possible that the information concerning PHSM was effective for Portuguese university students, empowering them to “determine relevance” (i.e., decide whether the information is applicable, apply the found information into daily life and use the information to make decisions about health) in daily life in a shorter time.

Searching for personal protective measures was associated with a higher likelihood of achieving sufficient “evaluating the reliability” of health information in a shorter time. It is likely that searching for personal protective measures enabled university students to extract information, develop meaning from different sources, and act independently on new information, thus achieving a sufficient “evaluating reliability” (i.e., decide whether the information is reliable or not, decide whether the information is written with commercial interests and check different websites to see whether they provide the same information) in a shorter time.

The subscales of appraising and applying health information (“evaluating reliability” and “determining relevance”) are considered more complex skills, also named critical health literacy [40]. This indicates that in the context of a pandemic and infodemic [5], searching for PHSM—notably personal protective measures related to coronavirus and COVID-19—besides the likelihood of achieving sufficient health literacy skills, also has an advantage on the more complex competencies. University students who searched for these measures might consider this information reliable, which might increase their personal credibility compared to those who did not search for the personal protective measures related to coronavirus and COVID-19. From the involvement theory standpoint [41], critical health literacy may motivate individuals to seek and appraise the quality of information pertaining to coronavirus and COVID-19. It seems that the process involved in the functional and interactive DHL, as reported in a previous study [42], is considered less influential than critical DHL. Intervention programs aiming at improving DHL may affect the online information itself and influence future actions [43].

Compared to those who never searched for information on environmental, economic and psychosocial issues, those who did ever search are more likely to determine the relevance of the content related to new coronavirus and COVID-19 on internet sources such as forums or social media. Although COVID-19 has a tremendous effect on mental health [14,44,45], a reasonably low percentage indicates searching for information on how to deal with psychological stress (please see Supplementary Materials Figure S1). As this study was conducted in the early stage of the pandemic in Portugal, it might have attenuated the concerns related to coronavirus and COVID-19.

This study is not without limitations. First, we underscore its cross-sectional design, precluding the establishment of causality among the variables. Second, we used a convenience sample of university students and an online questionnaire, excluding those without Internet access at the time of data collection. However, in Portugal, in 2020, there were 396,909 students in higher education, 54.1% of whom female [46], and this study comprehensively contributes to discussing important issues of PHSM searching and DHL among Portuguese university students. Third, we cannot generalize the results to the general population, because students are considered highly-educated and with access to computers/Internet. It is possible that these variables might have a “mixing of effects” [47,48], wherein the effects of the exposure (i.e., searching for PHSM) on the outcome (i.e., DHL) are mixed in with the effects of these additional factors (i.e., access to computer/Internet). However, since all the participants are university students, we have no reason to hypothesize that access to computer/Internet could result differently in a distortion of the found associations. Fourth, the self-perceived and remembered search queries about PHSM are reported, and the actual behavior may differ from this. Finally, we centered on the PHSM that university students searched, lacking information on the timeline of students’ searches and the amount of time they spent searching.

The study has important strengths. First, we emphasize the novelty of the research in the current pandemic and infodemic. This study highlights the associations between the topics searched for by university students and their DHL, contributing to the development and implementation of intervention programs focused on preparedness for future infodemics, tackling the infodemic, fostering health literacy, health promotion and prevention of COVID-19 [49]. Second, the current study is integrated into a DHL network related to COVID-19, allowing the comparison of results in different countries. Finally, the analyses accounted for essential confounders, considered important determinants of DHL.

Health information related to coronavirus and COVID-19 considered trustworthy and reliable is crucial for evidence-based practice, and citizens act accordingly with the best evidence. In the current information (or infodemic) age [8], there is a wide range of digital health information, some unreviewed or of questionable quality. The skills needed to search, select, appraise, communicate and integrate health information into daily life require health literacy, particularly DHL. Furthermore, the analysis of the online information queries (the “demand” side), what is published on websites, official portals and other engines (the “supply” side) [8] is considered crucial to identify gaps between what is known (evidence-based) and what is communicated (information reality). Enlargement in this gap increases the likelihood of applying harmful health practices and the risk of further spreading SARS-CoV-2.

## 5. Conclusions

This study reports a starting point in analyzing the trends of online information queries about PHSM on achieving sufficient DHL. Considering the political and economic diversity across countries, strategies to boost public confidence in pandemic response strategies may be anchored while respecting local singularities and integrating the “demand” side of information, along with the “supply” side with policymakers and end-users. With large-scale studies, future research is needed to analyze how online behavior influences and is influenced by current and future pandemics and the infodemic.

## Figures and Tables

**Table 1:** Descriptions of participants.

	All	Females	Males	*p*
Participants	3084	75.7	23.9	
Age [mean (SD)]	24.2 (7.5)	23.8 (7.0)	25.5 (8.9)	≤0.001 ^a^
Course [n (%)]				
Engineering sciences	386 (14.7)	195 (9.8)	188 (29.7)	≤0.001 ^b^
Humanities	145 (5.5)	109 (5.5)	34 (5.4)	
Exact sciences|natural|other	253 (9.6)	193 (9.7)	57 (9.0)	
Health sciences	886 (33.7)	752 (37.9)	134 (21.2)	
Social sciences|Psychology|Education	960 (36.5)	737 (37.1)	220 (34.8)	
Degree of study [n (%)]				
Bachelor	1331 (50.7)	1047 (52.8)	278 (43.9)	≤0.001 ^b^
Master (integrated)	544 (20.7)	373 (18.8)	168 (26.5)	
Post-graduation and master	543 (20.7)	407 (20.5)	135 (21.3)	
Doctorate	209 (8.0)	157 (7.9)	52 (8.2)	
Subjective social status [n (%)]				
Below median	1345 (51.4)	1020 (51.4)	325 (51.5)	0.967 ^b^
Median and above	1270 (48.6)	964 (48.6)	306 (48.5)	

^a^ Results from t test or Mann–Whitney. ^b^ Results from Chi squared test.

**Table 2:** Digital health literacy related to COVID-19 and socio-demographics of university students.

	Information Search		Adding Self-Gen. Content		Determining Relevance		Evaluating Reliability	
	Limited	Sufficient	Limited	Sufficient	Limited	Sufficient	Limited	Sufficient
	N (%)	N (%)	N (%)	N (%)	N (%)	N (%)	N (%)	N (%)
Participants	999 (54.7)	827 (45.3)	1308 (72.1)	505 (27.9)	993 (54.4)	832 (45.6)	970 (53.2)	854 (46.8)
**Sex**	n.s.		χ^2^ (1) = 7.204, *p* = 0.007		χ^2^ (1) = 4882, *p =* 0.027		n.s	
Male	233 (52.0)	215 (48.0)	299 (67.2)	146 (32.8)	223 (49.9)	224 (50.1)	245 (54.8)	202 (45.2)
Female	766 (55.6)	612 (45.3)	1009 (73.8)	359 (26.2)	770 (55.9)	608 (44.1)	725 (52.7)	652 (47.3)
**Course**	n.s.		n.s.		n.s.		n.s.	
Engineering sciences	126 (50.6)	123 (49.4)	183 (73.5)	66 (26.5)	134 (53.8)	115 (46.2)	146 (58.6)	103 (41.4)
Humanities	48 (51.6)	45 (48.4)	66 (71.7)	26 (28.3)	47 (50.5)	46 (49.5)	47 (51.1)	45 (48.9)
Exact sciences|natural|other	87 (51.5)	82 (48.5)	125 (74.4)	43 (25.6)	88 (52.1)	81 (47.9)	82 (48.8)	86 (51.2)
Health sciences	356 (55.4)	287 (44.6)	468 (73.7)	167 (26.3)	354 (55.1)	289 (44.9)	331 (51.5)	312 (48.5)
Social sciences|Psychology|Education	380 (57.1)	286 (42.9)	463 (35.5)	201 (30.3)	365 (54.9)	300 (45.1)	361 (54.2)	305 (45.8)
**Degree of study**	n.s.		n.s.		n.s.		n.s.	
Bachelor	511 (55.5)	409 (44.5)	652 (71.4)	261 (28.6)	499 (54.2)	421 (45.8)	501 (54.5)	419 (45.5)
Master (integrated)	196 (53.8)	168 (46.2)	267 (74.2)	93 (25.8)	204 (56.2)	159 (43.8)	203 (55.9)	160 (44.1)
Post-graduation and master	216 (57.3)	161 (42.7)	272 (72.5)	103 (27.5)	205 (54.4)	172 (45.2)	191 (50.7)	186 (49.3)
Doctorate	76 (46.1)	89 (53.9)	117 (70.9)	48 (29.1)	85 (51.5)	80 (48.5)	75 (45.7)	89 (54.3)
**Subjective Social Status**	n.s.		n.s.		n.s.		χ^2^ (1) = 6.598, *p* = 0.010	
Below median	534 (56.8)	464 (52.4)	690 (73.9)	244 (26.1)	530 (56.4)	410 (43.6)	527 (56.1)	413 (43.9)
Median and above	406 (43.2)	421 (47.6)	617 (70.3)	261 (29.7)	462 (54.4)	422 (47.7)	442 (50.1)	441 (49.9)

Sample sizes vary, according to missing data, in students that did not answer the full questionnaire. *p* value results from Chi squared test.

**Table 3:** Associations between online information about public health and social measures and digital health literacy related to COVID-19.

Digital Health Literacy Related with COVID-19								
Public Health and Social Measures	Information Search		Adding Self-Gen. Content		Evaluating Reliability		Determining Relevance	
	Unadjusted	Adjusted	Unadjusted	Adjusted	Unadjusted	Adjusted	Unadjusted	Adjusted
Personal protective measures	1.1 (0.8; 1.4)	1.2 (0.9; 1.4)	1.0 (0.7; 1.4)	1.1 (0.9; 1.5)	**1.4 (1.1; 1.9)**	**1.4 (1.1; 1.7)**	**1.7 (1.3; 2.3)**	**1.5 (1.2; 1.8)**
Surveillance and response measures	1.3 (1.0; 1.9)	1.3 (1.0; 1.6)	1.3 (0.9; 1.9)	1.3 (0.9; 1.8)	1.4 (1.0; 1.9)	1.3 (1.0; 1.7)	**1.7 (1.2; 2.4)**	**1.4 (1.1; 1.9)**
Environmental, economic and psychosocial measures	1.0 (0.8; 1.3)	1.1 (0.9; 1.39	1.2 (0.9; 1.5)	1.2 (0.9; 1.5)	1.1 (0.9; 1.4)	1.2 (1.0; 1.4)	**1.3 (1.0; 1.6)**	**1.2 (1.0; 1.4)**

Results from Cox hazard regression models; hazard ratio (95% confidence interval). Model adjusted for sex, age, subjective social status, course and degree of study. Bold: *p* ≤ 0.05.

## Data Availability

The data that support the findings of this study are available on request from the corresponding author (R.R.).

## Supplementary Materials

The following supporting information can be downloaded at: https://www.mdpi.com/article/10.3390/ijerph191912320/s1, Figure S1: Internet search queries (frequencies in %).
