# Supplementary figures and images for: Infodemic Preparedness and COVID-19: Searching about Public Health and Social Measures Is Associated with Digital Health Literacy in University Students
Source: Int J Environ Res Public Health. 2022 Sep 28;19(19):12320. doi: 10.3390/ijerph191912320 (PMC9566089; doi:10.3390/ijerph191912320)

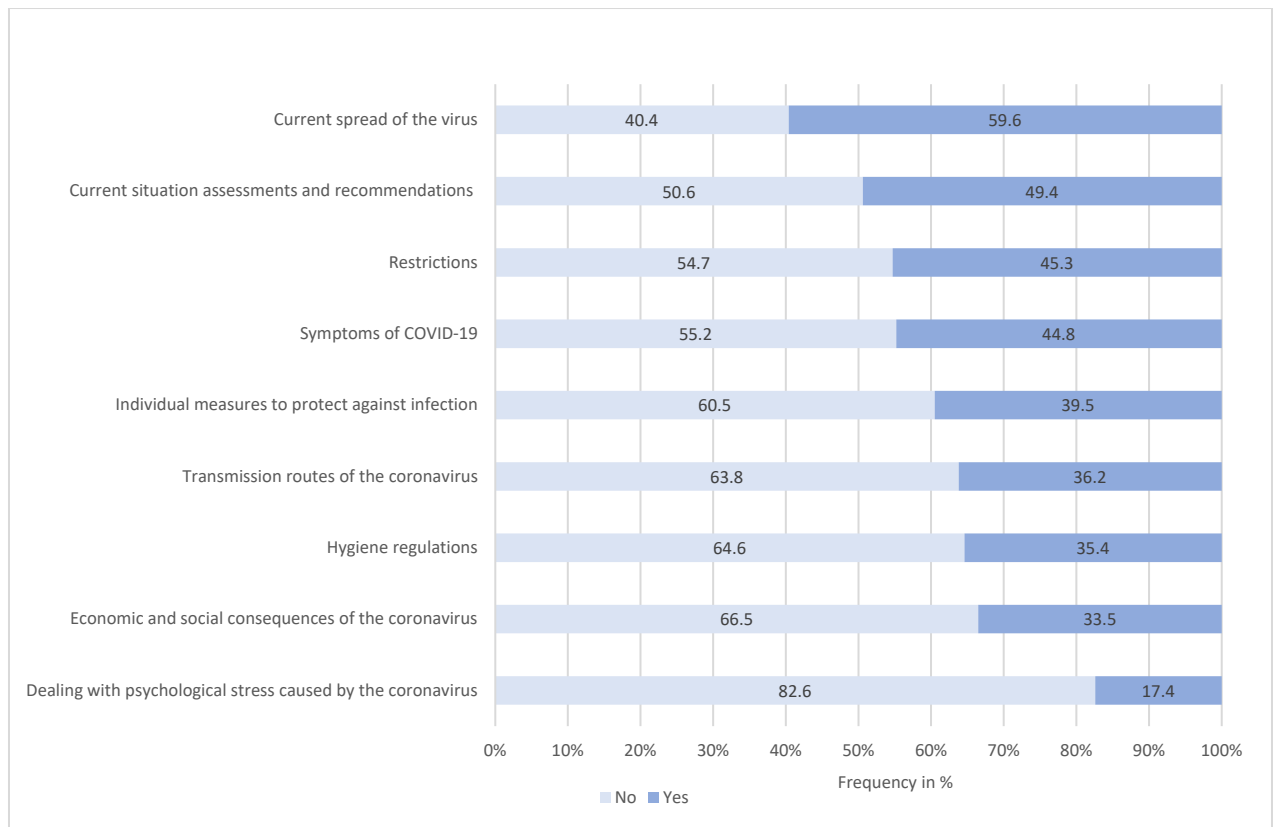

Figure S1. Internet search queries (frequencies in %).

Supplement: Supplementary file 1 [file ijerph-19-12320-s001.zip › ijerph-1873118-supplementary.pdf]
